# Wegener’s granulomatosis in which rheumatoid factor was useful for evaluating the disease status: a case report

**DOI:** 10.4076/1757-1626-2-6323

**Published:** 2009-06-26

**Authors:** Hideto Oshita, Hiromi Matsumoto, Teppei Hoshino, Keitaro Omori, Naoki Okamoto, Yukikazu Awaya

**Affiliations:** 1 Department of Respiratory Medicine, Chugoku Rosai General Hospital1-5-1, Hiro-tagaya, Kure-shi, Hiroshima 737-0193Japan; 2 Department of Internal Medicine, Kita-Kyusyu General Hospital5-10-10, Yukawa, Kokura-minami-ku, Kitakyusyu-shi, Fukuoka 800-0257Japan

## Abstract

**Introduction:**

Accurate evaluation of disease status is very important in treatment of Wegener's granulomatosis

**Case presentation:**

A 73-year-old Japanese man presented with chronic sinusitis and otitis media. He was admitted to our hospital because of bilateral lung nodules. Cytoplasmic antineutrophil cytoplasmic antibody was negative but his rheumatoid factor was high. He was diagnosed with limited Wegener's granulomatosis and received remission induction therapy. His serum rheumatoid factor level correlated with the Wegener's granulomatosis state when he experienced a severe infection and recurrence due to Wegener's granulomatosis.

**Conclusion:**

We describe a case of Wegener's granulomatosis in which rheumatoid factor was helpful for evaluating the therapeutic effect.

## Introduction

Wegener's granulomatosis (WG) is a necrotizing granulomatous vasculitis with multisystem involvement. Prognosis, which was once fatal, has been dramatically improved by treatment with immunosuppressive therapy. However, an accurate evaluation is necessary to design a treatment regimen. We describe a case of cytoplasmic antineutrophil cytoplasmic antibody (c-ANCA) negative WG in which rheumatoid factor (RF) was useful for evaluating the disease status.

## Case presentation

A 73-year-old Japanese man presented with a one year history of chronic sinusitis and otitis media that was unresponsive to otorhinolaryngological therapy. Because bilateral lung nodules were revealed by chest radiography, he was admitted to our hospital. He had a history of pneumoconiosis and cerebral infarction. Physical examination on admission revealed fever (36.8°C), pulse of 80 beats per minute, and blood pressure of 118/70 mmHg. The abnormal findings on physical examination were remarkable bilateral hearing loss, a saddle-nose deformity, and rhonchi in the middle left lung field.

Laboratory tests revealed the following: white blood cells count (WBC) of 7,900/mm^3^ (68.5% neutrophils, 0.6% eosinophils, 19.5% lymphocytes); hematocrit, 38.3%; hemoglobin, 12.7 g/dL; platelet count, 501,000/mm^3^; C-reactive protein (CRP), 16.2 mg/dL (normal, < 0.3 mg/dL); and erythrocyte sedimentation rate (ESR), 108 mm/hr. Laboratory tests for renal function, including urinalysis, were normal. c-ANCA was negative but RF was high (154 IU/ml, latex agglutination assay [normal, < 20 IU/mL]).

**Figure 1.**
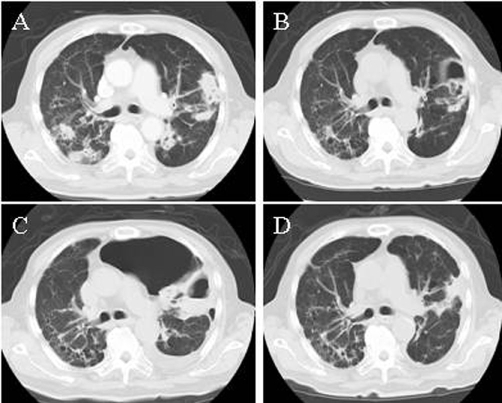
Chest computed tomography scans. Chest computed tomography scans on admission **(A)**, 3 **(B)**, 6 **(C)**, and 12 weeks **(D)** after the onset of the induction therapy.

**Figure 2.**
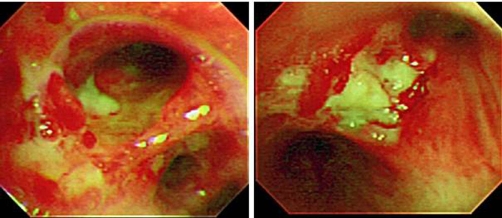
Bronchoscopy revealed multiple bronchial ulcers with pus.

Chest radiography and computed tomography (CT) showed multiple nodules and infiltrates with cavitations in the bilateral lung (Figure 1A). A bronchoscopic examination revealed multiple bronchial ulcers with pus (Figure 2). Biopsies of the bronchial and nasal mucosa showed nonspecific acute and chronic inflamed tissue with multinucleated giant cells; however, there was no evidence of malignancy or tuberculosis in these tissues. WG (limited disease) was diagnosed despite negative c-ANCA because our case met the American College of Rheumatology Diagnosis Criteria of 1990 for WG [1].

**Figure 3.**
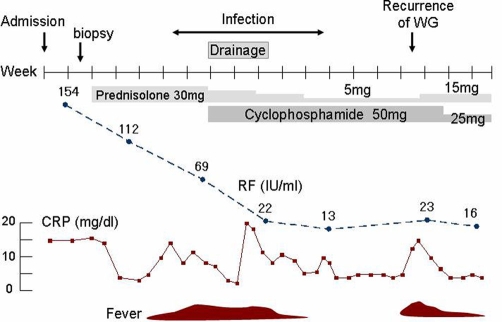
Clinical course.

He received remission induction therapy with prednisolone (PSL) 30 mg/day and trimethoprim-sulfamethoxazole (1:5) 960 mg/day, which improved his symptoms and laboratory test abnormalities. In particular, there was a decrease in serum RF level (Figure 3). CT scans revealed that the multiple lung nodules reduced in size or had changed to cystic lesions (Figure 1B).

Four weeks after the onset of therapy, his condition was complicated by severe pyothorax and pneumothorax (Figure 1C). He was successfully treated with antibiotics (linezolid and meropenem) and tube thoracostomy drainage. Although CRP, WBC, and ESR were elevated during the infection, serum RF level continued to decrease and CT scans showed a reduction in the size of the multiple lung nodules. We therefore realized that we were controlling WG. Because we feared deterioration in patient condition due to infection, cyclophosphamide 50 mg/day was added to the induction therapy and PSL was rapidly tapered to a maintenance dose of 5 mg/day.

Fourteen weeks after the onset of therapy, he complained of fever again, although there were no signs of infection. A CT scan revealed an increase in the size of the lung nodules and slight elevation in serum RF level, suggesting recurrence of WG, and the PSL was increased to 15 mg/day. Consequently, his fever abated and serum RF normalized.

## Discussion

WG is the most common of the ANCA-associated vasculitis diseases. It is clinically characterized by a triad of upper airway involvement, lower respiratory tract involvement, and glomerulonephritis [2]. Our patient showed negative c-ANCA but was diagnosed with WG based on clinical symptoms, imaging studies, and the microscopic findings that were not in contradiction with WG.

C-ANCA is highly sensitive (90-95%) in active, systemic WG, with a specificity of approximately 90% and is usually but not always correlated with the disease state [3,4]. However, c-ANCA was negative in our case. CRP, ESR, and WBC are not, of course, specific markers for WG and their variation can be caused by various stressors such as infection, drugs, trauma, among others.

In WG patients with a negative c-ANCA or in whom c-ANCA is not correlated with the disease state, the evaluation of disease status and therapeutic effect are difficult because the evaluation of WG are based on symptoms, imaging studies, and non-specific inflammatory markers.

Antibodies to altered γ-globulin, so-called RF, occur in approximately 70% of patients with rheumatoid arthritis (RA) [5,6]. A high RF level helps confirm the diagnosis of RA. In addition, RF serum level can be influenced by treatment and often falls as inflammatory activity decreases. RF is not specific for RA and is found in many diseases (e.g.; chronic infections, hepatitis, sarcoidosis, granulomatous diseases, subacute bacterial endocarditis). Noritake et al. [7] reported that an elevated RF was observed in about half of WG cases.

In this case, we predicted that a high RF level was useful for evaluating the disease state and measured it regularly. As expected, RF serum level correlated with the WG state. In particular, when the patient condition was complicated by a severe infection, the decreasing RF level was extremely helpful when we decided to taper the PSL.

Even if c-ANCA is positive, there are certain limitations in evaluating the WG state by c-ANCA. In a series of 106 patients, Kerr et al. [4] reported that c-ANCA titer temporally correlated with the WG state in only 64% of patients. In conclusion, there is no definitive marker for WG and careful and comprehensive observation of symptoms, laboratory tests, and imaging studies are most important in all cases. Nevertheless, in cases of WG with a high RF level, follow-up of this marker is thought to be of value for evaluating the disease status. Therefore, further study of WG cases with a high RF level may aid in determining the usefulness of this classical marker.

## Conclusions

We describe a case of WG in which RF correlated with the disease status and was useful for evaluating the therapeutic effect.

## Abbreviations

C-ANCA: Cytoplasmic antineutrophil cytoplasmic antibody
CRP: C-reactive protein
CT: Computed tomography
ESR: erythrocyte sedimentation rate
PSL: Prednisolone
RA: Rheumatoid Arthritis
RF: Rheumatoid factor
WG: Wegener's granulomatosis
WBC: White blood cells counts
